# Supplementary material for: Online informal learning community for interpreter training amid COVID-19: A pilot evaluation
Source: PLoS One. 2022 Nov 3;17(11):e0277228. doi: 10.1371/journal.pone.0277228 (PMC9632899; doi:10.1371/journal.pone.0277228)
Supplement: S1 Appendix — (DOCX) [file pone.0277228.s001.docx]

**Supporting Information**

**S1 Appendix. Satisfaction Survey for the Pilot Project**

| **No.** | **Dimensions** | **Items** |
| --- | --- | --- |
| 1 | Interaction in learning | The amount of interaction between you and your instructor and peers |
| 2 |  | The quality of interaction between you and your instructor and peers |
| 3 |  | The cooperation between you and your peers during learning. |
| 4 | Contents of the informal learning project | The manner in which the tasks of the learnig project were distributed |
| 5 |  | The logical organization of the leanring content. |
| 6 |  | The flexibility given to you to complete the tasks |
| 7 |  | The manner in which guidelines were given on the completion of tasks |
| 8 |  | The supportive learning materials provided to you |
| 9 |  | The extra learning resources provided to you |
| 10 | Tasks within the informal learning project | The format of the different tasks |
| 11 |  | The learning value of the tasks |
| 12 |  | The options available to you to hand in tasks |
| 13 |  | The quality of the feedback provided on graded tasks |
| 14 |  | Access to your performance rating during the course |
| 15 | Teaching and Learning style | The teaching and learning style you experienced in the project |
| 16 |  | The assistance given by the instructor in completing the course successfully |
| 17 |  | The instructor in terms of his devotion to the course |
| 18 |  | The accommodation of your approach to learning in the way this learning project is designed |
| 19 | Learning outcomes | The increase in your interpreting competence as a result of this project |
| 20 |  | The increase in your confidence in using the knowledge to solve interpreting problems as a result of this project |
| ***These items were assessed using a 5-point Likert scale with an anchor value of 1 for “strongly disagree” and 5 for “strongly agree”.** | | |
