# Supplementary material for: Online informal learning community for interpreter training amid COVID-19: A pilot evaluation
Source: PLoS One. 2022 Nov 3;17(11):e0277228. doi: 10.1371/journal.pone.0277228 (PMC9632899; doi:10.1371/journal.pone.0277228)
Supplement: S2 Appendix — (DOCX) [file pone.0277228.s002.docx]

**S2 Appendix. Results of the Satisfaction Survey**

|  | Sat.2020.Dec | Sat.2021.Apr | Sat.2021.Dec | Sat.2022.Mar |
| --- | --- | --- | --- | --- |
| Student1 | 68 | 78 | 84 | 81 |
| Student2 | 68 | 77 | 85 | 84 |
| Student3 | 67 | 73 | 81 | 81 |
| Student4 | 67 | 75 | 83 | 82 |
| Student5 | 66 | 72 | 79 | 83 |
| Student6 | 71 | 71 | 82 | 83 |
| Student7 | 69 | 75 | 80 | 80 |
| Student8 | 71 | 70 | 83 | 83 |
| Student9 | 71 | 76 | 83 | 82 |
| Student10 | 72 | 72 | 82 | 76 |
| Student11 | 68 | 77 | 81 | 82 |
| Student12 | 70 | 73 | 84 | 86 |
| Student13 | 66 | 74 | 84 | 81 |
| Student14 | 67 | 74 | 86 | 81 |
| Student15 | 72 | 75 | 83 | 80 |
| Student16 | 69 | 74 | 85 | 82 |
| Student17 | 72 | 71 | 83 | 81 |
| Student18 | 75 | 77 | 82 | 81 |
| Student19 | 72 | 73 | 83 | 83 |
| Student20 | 72 | 74 | 82 | 84 |
| Student21 | 69 | 74 | 83 | 84 |
| Student22 | 71 | 73 | 78 | 82 |
| Student23 | 71 | 72 | 81 | 83 |
| Student24 | 62 | 76 | 82 | 79 |
| Student25 | 71 | 78 | 81 | 81 |
| Student26 | 70 | 73 | 81 | 81 |
| Student27 | 72 | 70 | 87 | 82 |
| Student28 | 68 | 76 | 84 | 83 |
| Student29 | 71 | 75 | 81 | 79 |
| Student30 | 70 | 72 | 83 | 84 |
| Student31 | 71 | 77 | 83 | 81 |
| Student32 | 66 | 77 | 85 | 85 |
| Student33 | 71 | 68 | 80 | 82 |
| Student34 | 69 | 73 | 83 | 79 |
| Student35 | 72 | 71 | 80 | 81 |
