# Supplementary material for: Online informal learning community for interpreter training amid COVID-19: A pilot evaluation
Source: PLoS One. 2022 Nov 3;17(11):e0277228. doi: 10.1371/journal.pone.0277228 (PMC9632899; doi:10.1371/journal.pone.0277228)
Supplement: Attachment — Submitted filename: PLOS ONE review.NA.docx [file pone.0277228.s003.docx]

Here are some small edits that the writers should make to the paper that has been reviewed and attached. However, the work's quality is good, and after a short edit, it can be accepted for publishing, barring a few minor corrections. This is a well-written piece, and I applaud the authors for their efforts. The introduction and method sections need some small tweaks, but all other sections are well-organized and explained.

- extremely well-structured writing. It would be fantastic if the authors briefly described informal learning in section 1.1 before beginning to describe it in detail in section 1.2.
- Methodology

The authors did a good job of explaining their research methodology, but in section 2.3, lines 170, 171, and 172, I would advise double-checking the sources of the qualitative and quantitative data because the authors only mentioned the qualitative, and I think line 171 will actually be presenting quantitative data as a numeric presentation.
