# Supplementary material for: Online informal learning community for interpreter training amid COVID-19: A pilot evaluation
Source: PLoS One. 2022 Nov 3;17(11):e0277228. doi: 10.1371/journal.pone.0277228 (PMC9632899; doi:10.1371/journal.pone.0277228)
Supplement: Attachment — Submitted filename: Responses to the reviewers.docx [file pone.0277228.s004.docx]

Dear Assoc. Prof. Omar Khraisat,

Greetings,

Thanks for being the editor of the manuscript under the consideration of PLOS ONE entitled *Online Informal Learning Community for Interpreter Training Amid COVID-19: A Pilot Evaluation* (**PONE-D-22-21532**). After receiving the reviewers’ comments, the authors began a thorough amendments and revision of the original submission.

We would like to extend the warmest thanks to both of the reviewers for their valuable comments and constructive suggestions. The manuscript has been revised in accordance with these comments. Changes made in response to the reviewer are described as follows.

**Response to the Comments from Reviewer #1:**

First, in regard to the comments and suggestion which only needs a relatively small proportion of contents to be revised, our responses are as follows:

Line 11: Term “reviewed” is the author referring to assessment or the term “viewed” referring to perception. The subsequent statement seemingly refers to perception vs assessment and the most appropriate term may be “viewed”

Response: Thanks for the suggestion. The word “viewed” was used in the revised manuscript.

Line 18,135, 312: “N” usually refers to a population size while “n” refers to sample size. Thirty-six is in reference to sample size and thus lower case “n” should be used.

Response: Thanks for pointing out the issue. The distinction between “N” and “n” was made in the revised manuscript.

Line 33: Lack of coherence between the succeeding statement. The new emerging issues rather than the amplification of underlying issues should follow the first statement.

Response: Thanks for pointing out the issue. The sentence order was rearranged to avoid the ambiguity in the original manuscript. Please see LINE 33-36 for the amendments.

Line 78, 103, 468 etc: from the programmatic perspective, I would use impact of the .. rather than the effect.

Response: Thanks for the suggestion. Impact was used to replace effect in the manuscript in all similar occasions.

Line 111, 133; I feel it the rest of the manuscript does not reflect the measurement of “effectiveness” would substitute it with “impact”. Or you may want to provide operational definition of “effectiveness” Additionally, use of “experimental informal…” I find it misleading. When I read that, as a reader, I am looking for rigorous quantitative methodology with comparative groups which is not the case. I would leave it to the “pilot” project as used in some instances. Line 113 and 114, are more appropriate.

Response: Thanks. We agree that for qualitative-dominant research as the paper, the word “effectiveness” was misleading to the readers. “Impact” was used to replace effectiveness in the manuscript in all similar occasions.

Line 171: Review “… qualitative data as numeric….”, this should be quantitative

Response: Thanks for pointing out the typo. The correction was made accordingly.

Line 173-177: Abbreviate Focus group discussion (FGD) and use acronym subsequently. Did you have any gender considerations in the FGDs? How many FGDs were conducted in total and what was the stopping rule?

Response: Thanks for suggesting the abbreviation. We have adopted your suggestion in the manuscript. Gender issues were not considered in the FGD, however, as in many language education settings, the population was female dominant. Regarding the rounds of FGD conducted in the research, please kindly refer to LINE 178-180 for the description about the total number of FGD sessions conducted and the stopping rule for the FGD. An excerption was as follows:

*In total, eight sessions of FGD were conducted until maturity of the pilot project and saturation of data collection were reached in accordance with the views from expert panel members and the authors.*

Line 184-191: It is not clear whether snow balling sampling was done both qualitative and quantitative inquiries.

Response: Thanks for pointing out. The sampling method was used for both strands of research. As the participants were the student involved in the informal learning project. All corresponding data collection and analysis were conducted with them as the participants.

Line 192-194: The content in this paragraph seems to miss a heading as the content does not match well with the tittle participant on line 183.

Response: Thanks for the suggestion. In the revised manuscript, the paragraph now falls into a separate section entitled “Ethics”. Please refer to section 2.6 for details.

Line 198: This takes account of FGDs conducted at the end of the semester and yet earlier on line 176 and 177, this took account of FGDs at the beginning and end of the semester

Response: Thanks for the pointing out the issue. The FGD were conducted at both the beginning and end of the semester. In the updated manuscript, all relevant information were checked and revised for internal consistency.

Line 204: It is not clear of what media participants checked. Did they check the recording or the transcript or both?

Response: The media checked by participants involved both the audio recording and the transcript verbatim.

Line 206-210: Who was involved in this process and if they are investigators, did you examine your own role, potential bias and influence during analysis and selection of data for presentation? It is also good practice to provide a code book as a supplementary material

Response: Thanks for the pointing it out. We agree that this is very important for the qualitative research within the paper. Please check LINE 212-214 for the amended information regarding the issue.

As to the codebook, a sample codebook was added to the manuscript as TABLE 4. The table and relevant descriptions could be found in the manuscript in LINE 211-212.

Line 211-215: What were the measurements for satisfaction and what are the major learning artifacts that were tracked.

Response: Thanks. We agree that the clarification was important for the manuscript. The measurement of satisfaction was with a survey adopted from the instrument used in previous studies. As to the contents of learning artifacts to be studied, the most important was students’ performance in interpreting tasks and their notetaking (which was a very important learning artifact in an interpreter training setting). The details could be found in the revised manuscript at LINE 181-184 and LINE 224-225.

General: Most of the quotes don’t have source like cited on line 309

Response: Thanks for the pointing it out. We agree that inconsistency in citing the quotes was causing trouble. In the updated manuscript, all sources were amended. Please refer to the quotes.

Line 222, 233: Mention the pilot project

Response: Thanks for the pointing it out. However, we were not very clear about the suggestion of “mentioning the pilot project”. In the revision, we changed the title of section 3.1 from “Strength” into “Strength of the Pilot Project” for clarity. If the solving does not solve the issue you have suggested, please let us know.

Line 248: Consensus vs consent,

Response: Thanks for the pointing out the typo. It has been corrected, and we are sorry for the mistake.

Line 249-250: Observation was not mentioned in the methodology; the interpretation seems out of context as the cited quote does not illustrate that

Response: Thanks for the comments. We have amended “observation” as a supportive research method in the methodology. Please refer to LINE 184-185 of the updated manuscript for details.

In regard to the out of context quote from the participating student, the quote is now substituted by a quote from a lecturer involved in the project based on her observations of student’s involvement and activities within the project. Please refer to LINE 268-271 for details.

Line 260-261: italicize

Response: Thanks for the pointing out the issue. It has been reformatted accordingly.

Line 265: First time to come across project-based learning. Earlier in the methods, you mentioned about three pillars of which this was not one of them, even the conceptual diagram does not reflect this. Clarify.

Response: Thanks for the pointing out the issue. We agree that is a very important one and is causing inconsistency between different sections of the paper.

We have replaced “project-based learning” with “experiential learning” which is more appropriate for the study, especially in line with the conceptual model of the pilot project. However, we would like to point out that in the original design, project-based learning was actually the main approach to implement “experiential learning”, given the nature of interpreting education and common practice within the discipline.

In a nutshell, we are indebted to the reviewer for pointing out the issue as we were apparently taking it for granted in the original composition.

Line 282: Being an agricultural university--- is a potential identifier. I take note you would like to maintain anonymity. I take note that the author is from an agricultural university, whose name is mentioned and thus can link to the potential university where data was collected. On a second thought, this is good practice that other institutions of learning should learn from, I am curious why we are making the university has to be anonymous.

Response: Thanks for the pointing out the issue. We were not consistent in choosing to make the affiliations anonymized or exposed. After a series of repeated discussion between the authors, we would like to adopt the suggestions from the reviewer and make the information public.

Line 312: would recommend this format 24/36 (63.8%)

Response: Thanks for the suggestions, now the number were presented as “**63.8% (n=24)** of the students chose digital literacy”. Please kindly refer to LINE 329 for detail.

Line 314: the tittle of the subtheme should be consistent with the one in table 4

Response: Thanks for the pointing it out. And it is corrected as suggested.

Line 333: project vs experiment

Response: Thanks for the pointing out the typo. It has been corrected, and we are sorry for the mistake.

Line 338: Table 5 should be consistent with line 337 interms of number of sub themes; 3 vs 4. Terms rater vs assessor

Response: Thanks for the pointing it out. And it is corrected as suggested. As to the choice between rater and assessor, we choose to use “assessor” as the role played by trainers is not limited to rating student’s performance.

Line 375-379: does this fit well with the sub theme?

Response: Thanks for the pointing it out. The quote was removed from this section. And in the discussion, the doubts from students were treated as “unexpected findings” of the research.

Discussion on wards: Hard to read sections. Reader unfriendly

Response: Thanks for the comments. After systematic revision of the discussion section. We would attribute the “reader unfriendliness” to over-extended discussion related to decision-making and educational policies in interpreter training. In the revised manuscript, all discussion were based on the findings presented in the previous section. And all off-topic and out-of-context discussion were removed. Please kindly refer to and review the overhauled discussion section for the details.

Discussion: General comment: the discussion is insufficient. Any comparative evaluation with other studies

Response: Thanks for the comments. We agree that the discussion in the original manuscript was not enough. In the updated version, the part was totally rewritten. Now a comparative stance against existing literature was held by the authors. Please kindly refer to the revision for details.

Line 467: Though vs thought

Response: Thanks for the pointing out the typo. It has been corrected, and we are sorry for the mistake.

Implications: General comment: There are sections that best fit for result sections. Some of the statements are not consistent with methods and results.

Response: Thanks for the comments. We agree that some part in the implication and recommendations were not strictly based on the finding of the paper. Consequently, they were not tightly integrated with the context of the discussion. In the revision, we merged the implication and the recommendations. All claims are now based on the findings of the research, and explicit recommendations and implication were preferred.

Recommendations: General comment: Some of the recommendations are not consistent with the results.

Response: Thanks for the comments. As the recommendations and the implications were merged in the revision, please kindly refer to the response in the previous comments.

**Response to the Comments from Reviewer #2:**

**Comment 1:** It would be fantastic if the authors briefly described informal learning in section 1.1 before beginning to describe it in detail in section 1.2.

Response: Thanks for the comments. The suggestion was valuable, and we are happy to revise accordingly. In the revision, we added a background description of the current status of using informal learning online. Please kindly refer to LINE 41-43 for details.

**Comment 2:** The authors did a good job of explaining their research methodology, but in section 2.3, lines 170, 171, and 172, I would advise double-checking the sources of the qualitative and quantitative data because the authors only mentioned the qualitative, and I think line 171 will actually be presenting quantitative data as a numeric presentation.

Response: Thanks for the suggestion and complement. We have noticed the mistake and corrected accordingly.
